# Cocaine as an Antidote for Morphia

**Published:** 1885-07

**Authors:** 


### Cocaine as an Antidote for Morphia.

The *Wien Med. Blatter* reports on cocaine as an antidote for morphia, especially in cases of habitual use of the drug. The injection of one-seventh of a grain to a grain and a half of cocaine induces a condition very similar to that caused by a sudden stoppage of the morphia, but there is besides a peculiar sensation, as if the cocaine were seeking out the morphia in the body, and eliminating it by degrees. Griping then follows, as if from a drastic purgative, and copious thin stools ensue. The face becomes yellow, as in the abstinence cure, this passing off gradually as the cocaine gets the better of morphia in the organism. Cocaine may be used as a measure for the amount of morphia which is present in the body of the patient; as the symptoms become more intense, the more cocaine is given, until the morphia is entirely eliminated. It is suggested that a more pleasant mode of administration would be to give the two drugs together, and gradually to increase the cocaine while diminishing the morphia, in order to avoid the unpleasant symptoms of morphia hunger.

Dr. C. C. F. Gay sailed for Europe on the 16th ult., proposing to enjoy the pleasures of European travel until September. Although our distinguished friend seeks only rest and recreation, yet we expect that his professional ardor will lead him to the hospitals and schools, and we hope that our readers may have the benefit of his observation.

---

The preparations of Wm. F. Kidder & Co. have always given us satisfaction in their use. This is notably the case with *Hydroleine*, which is not only palatable, but is tolerated by the most delicate stomachs, when the pure oil or other emulsions are rejected.

---

Parke, Davis & Co. have prepared a number of antiseptic and disinfectant preparations, the composition of which is based upon the results of the most recent scientific researches. It is undeniable that many of the so-called disinfectants are valueless. This reliable firm of manufacturing chemists have, therefore, done the profession and public a service in putting upon the market a complete line of reliable disinfectants.

---

J. B. Lippincott & Co., 715 Market street, Philadelphia, have published a "clinical chart," designed by Dr. T. C. Wilson, which is by far the best we have seen for the convenient, accurate and permanent daily recording of cases. They are supplied in packets of fifty for half a dollar.

